# The Microbiome Protocols eBook initiative: Building a bridge to microbiome research

**DOI:** 10.1002/imt2.182

**Published:** 2024-03-19

**Authors:** Yunyun Gao, Kai Peng, Defeng Bai, Xiao‐Ye Bai, Yujing Bi, Anqi Chen, Baodong Chen, Feng Chen, Juan Chen, Lei Chen, Tong Chen, Wei Chen, Xu Cheng, Yanfen Cheng, Jie Cui, Jingjing Dai, Junbiao Dai, Zhaolai Dai, Ye Deng, Yi‐Zhen Deng, Wei Ding, Zhencheng Fang, Wei Fu, Hanbing Gao, Shaohua Gu, Xue Guo, Xuguang Guo, Dongfei Han, Lele He, Yatao He, Hui‐Yu Hou, Baolei Jia, Gengjie Jia, Shuo Jiao, Wei Jin, Feng Ju, Zhicheng Ju, Siyuan Kong, Canhui Lan, Bing Li, Da Li, Diyan Li, Jingdi Li, Meng Li, Qi Li, Qiang Li, Wen‐Jun Li, Xiaofang Li, Xuemeng Li, Yahui Li, You‐Gui Li, Zhibin Liang, Ning Ling, Fufeng Liu, Qing Liu, Shuang‐Jiang Liu, Hongye Lu, Qi Lu, Guangwen Luo, Hao Luo, Yuheng Luo, Hujie Lyu, Chuang Ma, Liyuan Ma, Tengfei Ma, Jinfeng Ni, Ziqin Pang, Xiaojing Qiang, Yuan Qin, Qingyue Qu, Chao Ran, Shuqiang Ren, Haitao Shang, Luyang Song, Linyang Sun, Weimin Sun, Liping Tang, Jian Tian, Kai Wang, Mengzhi Wang, Ming‐Ke Wang, Tao Wang, Xiao‐Yan Wang, Yao Wang, Yiwen Wang, Youshan Wang, Hailei Wei, Hong Wei, Zhong Wei, Tao Wen, Jiqiu Wu, Linhuan Wu, Linkun Wu, Jiao Xi, Bo Xie, Guofang Xu, Jun Xu, Shanshan Xu, Qing Xue, Liping Yan, Haifei Yang, Jun Yang, Junbo Yang, Ruifu Yang, Yalin Yang, Ying‐Jie Yang, Xiaofang Yao, Yanpo Yao, Salsabeel Yousuf, Ke Yu, Zhengrong Yuan, Zhilin Yuan, Dong Zhang, Tianyuan Zhang, Weipeng Zhang, Yunzeng Zhang, Zhaonan Zhang, Zhen Zhang, Zhi‐Feng Zhang, Shengguo Zhao, Wei Zhao, Maosheng Zheng, Ziqiang Zheng, Xin Zhou, Yuanping Zhou, Zhigang Zhou, Mo Zhu, Yong‐Guan Zhu, Haiyan Chu, Yang Bai, Yong‐Xin Liu

**Affiliations:** ^1^ Agricultural Genomics Institute at Shenzhen Chinese Academy of Agricultural Sciences Shenzhen China; ^2^ Jiangsu Co‐Innovation Center for Prevention and Control of Important Animal Infectious Diseases and Zoonoses, College of Veterinary Medicine Yangzhou University Yangzhou China; ^3^ Shenzhen Bay Laboratory Shenzhen China; ^4^ State Key Laboratory of Pathogen and Biosecurity Beijing Institute of Microbiology and Epidemiology Beijing China; ^5^ Bio‐Protocol Editorial Office China Bio‐Protocol Journal Beijing China; ^6^ Research Center for Eco‐Environmental Sciences Chinese Academy of Sciences Beijing China; ^7^ School of Stomatology Peking University Beijing China; ^8^ Institute of Medicinal Plant Development Chinese Academy of Medical Sciences Beijing China; ^9^ Department of Vascular Surgery, Fu Xing Hospital Capital Medical University Beijing China; ^10^ State Key Laboratory for Quality Ensurance and Sustainable Use of Dao‐di Herbs, National Resource Center for Chinese Materia Medica China Academy of Chinese Medical Sciences Beijing China; ^11^ Institute of Hydroecology Ministry of Water Resources & Chinese Academy of Sciences Wuhan China; ^12^ Nanjing Agricultural University Nanjing China; ^13^ The Institute of Infection and Health Research Fudan University Shanghai China; ^14^ Department of Medical Laboratory the Affiliated Huaian No.1 Hospital of Nanjing Medical University Huaian China; ^15^ China Agricultural University Beijing China; ^16^ State Key Laboratory for Conservation and Utilization of Subtropical Agro‐Bioresources, Guangdong Province Key Laboratory of Microbial Signals and Disease Control, Integrative Microbiology Research Centre South China Agricultural University Guangzhou China; ^17^ Ocean University of China Qingdao China; ^18^ Zhujiang Hospital Southern Medical University Guangzhou China; ^19^ Central South University Changsha China; ^20^ Center for Quantitative Biology and Peking‐Tsinghua Center for Life Sciences, Academy for Advanced Interdisciplinary Studies Peking University Beijing China; ^21^ Department of Clinical Laboratory Medicine, Guangdong Provincial Key Laboratory of Major Obstetric Diseases; Guangdong Provincial Clinical Research Center for Obstetrics and Gynecology The Third Affiliated Hospital of Guangzhou Medical University Guangzhou China; ^22^ School of Environmental Science and Engineering Suzhou University of Science and Technology Suzhou China; ^23^ Hunan University Changsha China; ^24^ School of Medicine, Model Animal Research Center (MARC) Nanjing University Nanjing China; ^25^ Xianghu Laboratory Hangzhou China; ^26^ National Key Laboratory of Crop Improvement for Stress Tolerance and Production, Shaanxi Key Laboratory of Agricultural and Environmental Microbiology, College of Life Sciences Northwest A&F University Yangling China; ^27^ Westlake University Hangzhou China; ^28^ Department of Ocean Science The Hong Kong University of Science and Technology Hong Kong China; ^29^ School of Life Science and Technology Wuhan Polytechnic University Wuhan China; ^30^ R‐Institute Co. Ltd. Beijing China; ^31^ Tsinghua Shenzhen International Graduate School Tsinghua University Shenzhen China; ^32^ Institute of Microbiology Chinese Academy of Sciences Beijing China; ^33^ Antibiotics Research and Re‐Evaluation Key Laboratory of Sichuan Province, Sichuan Industrial Institute of Antibiotics, School of Pharmacy Chengdu University Chengdu China; ^34^ University of Oxford Oxford UK; ^35^ Shenzhen Key Laboratory of Marine Microbiome Engineering, Institute for Advanced Study Shenzhen University Shenzhen China; ^36^ Institute of Applied Ecology Chinese Academy of Sciences Shenyang China; ^37^ School of Food and Biological Engineering Chengdu University Chengdu China; ^38^ School of Life Sciences Sun Yat‐Sen University Guangzhou China; ^39^ Center for Agricultural Resources Research, Institute of Genetics and Developmental Biology Chinese Academy of Sciences Shijiazhuang China; ^40^ Guangdong Medical University Dongguan China; ^41^ Zhejiang Academy of Agricultural Sciences Hangzhou China; ^42^ Guangdong Province Key Laboratory of Microbial Signals and Disease Control, Integrative Microbiology Research Centre South China Agricultural University Guangzhou China; ^43^ State Key Laboratory of Herbage Improvement and Grassland Agro‐ecosystems, Centre for Grassland Microbiome, College of Pastoral Agricultural Science and Technology Lanzhou University Lanzhou China; ^44^ College of Biotechnology Tianjin University of Science & Technology Tianjin China; ^45^ Zhejiang University Hangzhou China; ^46^ Children's Hospital of Chongqing Medical University Chongqing China; ^47^ Animal Nutrition Institute Sichuan Agricultural University Chengdu China; ^48^ Imperial College of London London UK; ^49^ Anhui Agricultural University Hefei China; ^50^ China University of Geosciences Wuhan China; ^51^ State Key Laboratory of Microbial Technology, Microbial Technology Institute Shandong University Qingdao China; ^52^ College of Agriculture Fujian Agriculture and Forestry University Fuzhou China; ^53^ Institute of Grassland Research Chinese Academy of Agricultural Sciences Hohhot China; ^54^ Institute of Genetics and Developmental Biology Chinese Academy of Sciences Beijing China; ^55^ Institute of Zoology Chinese Academy of Sciences Beijing China; ^56^ Feed Research Institute Chinese Academy of Agricultural Sciences Beijing China; ^57^ Shenzhen Medical Academy of Research and Translation Shenzhen China; ^58^ Henan Agricultural University Henan China; ^59^ Faculty of Biological and Environmental Sciences University of Helsinki Helsinki Finland; ^60^ Institute of Eco‐Environmental and Soil Sciences Guangdong Academy of Sciences Guangzhou China; ^61^ State Key Laboratory of Animal Nutrition and Feeding, Institute of Animal Sciences Chinese Academy of Agricultural Sciences Beijing China; ^62^ School of Marine Sciences Ningbo University Ningbo China; ^63^ Yangzhou University Yangzhou China; ^64^ Naval Medical Center of PLA Naval Medical University Shanghai China; ^65^ School of Life Sciences Taizhou University Taizhou China; ^66^ Institute of Plant Nutrition, Resources and Environment Beijing Academy of Agriculture and Forestry Sciences Beijing China; ^67^ Key Laboratory of Microbial Resources Collection and Preservation, Ministry of Agriculture, Institute of Agricultural Resources and Regional Planning Chinese Academy of Agricultural Sciences Beijing China; ^68^ The First Affiliated Hospital Sun Yat‐Sen University Guangzhou China; ^69^ Department of Genetics, University Medical Center Groningen University of Groningen Groningen The Netherlands; ^70^ Microbial Resource and Big Data Center, Institute of Microbiology Chinese Academy of Sciences Beijing China; ^71^ College of JunCao Science and Ecology Fujian Agriculture and Forestry University Fuzhou China; ^72^ College of Natural Resources and Environment Northwest A&F University Yangling China; ^73^ School of Life Sciences Central China Normal University Wuhan China; ^74^ Department of Civil and Environmental Engineering National University of Singapore Singapore Singapore; ^75^ Department of Gastroenterology, Clinical Center of Immune‐Mediated Digestive Diseases Peking University People's Hospital Beijing China; ^76^ Hefei University of Technology Hefei China; ^77^ Beijing Forestry University Beijing China; ^78^ Qingdao Agriculture University Qingdao China; ^79^ Key Laboratory of Urban Environment and Health, Institute of Urban Environment Chinese Academy of Sciences Xiamen China; ^80^ Tobacco Research Institute Chinese Academy of Agricultural Sciences Qingdao China; ^81^ Key Laboratory of Agro‐Ecological Processes in Subtropical Region, Institute of Subtropical Agriculture Chinese Academy of Sciences Changsha China; ^82^ Agro‐Environmental Protection Institute Ministry of Agriculture and Rural Affairs Tianjin China; ^83^ School of Environment and Energy Peking University Shenzhen Graduate School Shenzhen China; ^84^ State Key Laboratory of Tree Genetics and Breeding Chinese Academy of Forestry Beijing China; ^85^ Wuhan Benagen Technology Co., Ltd. Wuhan China; ^86^ Peking University Beijing China; ^87^ Southern Marine Science and Engineering Guangdong Laboratory (Guangzhou) Guangzhou China; ^88^ Tianjin Institute of Industrial Biotechnology Chinese Academy of Sciences Tianjin China; ^89^ College of Environmental Science and Engineering North China Electric Power University Beijing China; ^90^ College of Life Science and Technology Wuhan Polytechnic University Wuhan China; ^91^ College of Life Sciences Henan Normal University Xinxiang China; ^92^ Institute of Urban Environment Chinese Academy of Sciences Xiamen China; ^93^ State Key Laboratory of Soil and Sustainable Agriculture, Institute of Soil Science Chinese Academy of Sciences Nanjing China; ^94^ Peking‐Tsinghua Center for Life Sciences, College of Life Sciences Peking University Beijing China

## Abstract

The Microbiome Protocols eBook (MPB) serves as a crucial bridge, filling gaps in microbiome protocols for both wet experiments and data analysis. The first edition, launched in 2020, featured 152 meticulously curated protocols, garnering widespread acclaim. We now extend a sincere invitation to researchers to participate in the upcoming 2nd version of MPB, contributing their valuable protocols to advance microbiome research.

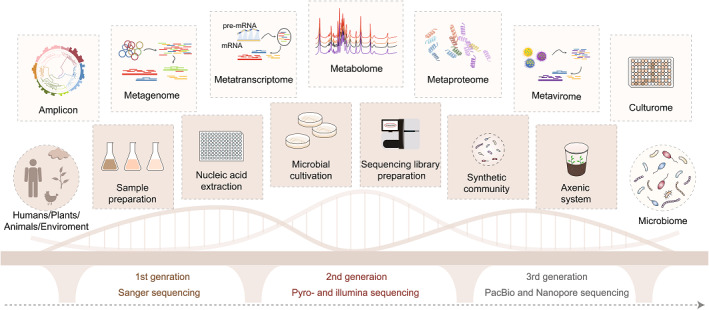

The microbiome is a research area that focuses on studying the omics of the microbe [1] with rapid development in the past few decades, making breakthroughs in understanding microbiological studies in humans [2], animals [3] and plants [4] as hosts, and the environments [5]. The concept of the microbiome has been extended to various fields. Thousands of microbiome articles are published every year, revolutionizing our conventional understanding of microbes in medicine, agriculture, and industry. At present, some standardized analysis software and protocols for microbiome studies have been developed. For example, QIIME2 [6] stands out as a popular integrated pipeline for amplicon sequencing data analysis, Minimum Information about any (x) Sequence (MIxS) serves as the standard for submitting microbiome sequences [7], Critical Assessment of Metagenome Interpretation [8] provides the standards of assessing metagenomics software. Despite these advancements in microbiome research, there remains a need for more standard methods for other types of microbiome data. In addition, few systematic and standard protocols for wet‐lab experiments and data analysis were published, which impeded the progress of experiments or analysis in this area. To address these issues, we initiated the Microbiome Protocols eBook (MPB, https://cn.bio-protocol.org/bio101/mpb), aimed at providing a comprehensive resource for standardized wet‐lab protocols in microbiome research.

The MPB was launched by Bio‐protocol office in China and WeChat's official account “meta‐genome,” one of the largest microbiome communities with more than 164,000 subscribers (by February 2024). The Bio‐protocol Journal offers peer‐reviewed and open‐access publications, at no cost. The MPB is designed to foster communications and collaboration between researchers and research teams, with the goal of summarizing, sharing, and disseminating the wet‐lab experiment protocols in the microbiome area. We anticipate that this project will bridge the gaps in microbiome protocols, addressing the challenges encountered in wet experiments and data analysis, while paving the way for accumulation of standard data for big data integrated analysis in the near future. In summary, the MPB is poised to essentially facilitate the progress of the microbiome area. All protocols are hosted in Bio‐101, a companion website of the Bio‐protocol Exchange. The project homepage link is https://bio-protocol.org/bio101/mpb.

The MPB encompasses a wide range of microbiome‐related protocol, including culturomics [9], amplicon [10], metagenome, metatranscriptome, metavirome, metaproteome, metabolome, microbiome, related molecular biology and microbiology experiments. It also covers the upstream and downstream experimental protocols and analysis (Figure 1A, Supporting Information: Table S1). According to the research objects, it mainly includes the microbiome in humans, animals, plants, and the environment (Figure 1A, B). This resource comprehensively covers a range of research methods, it mainly includes sample preparation, nucleic acid extraction, protein and metabolite extraction, sequencing library preparation, microbial culture and identification, synthetic community, axenic system, data analysis, and general microbiology experiments and analysis. MPB is designed to be easily accessible to all, providing convenient access through many channels such as Bio‐101, WeChat, Chinese Software Developer Network (CSDN, https://www.csdn.net/), and so forth. Its open‐access nature ensures that anyone can explore and benefit from the latest protocols and techniques in microbiome research.

**Figure 1:**
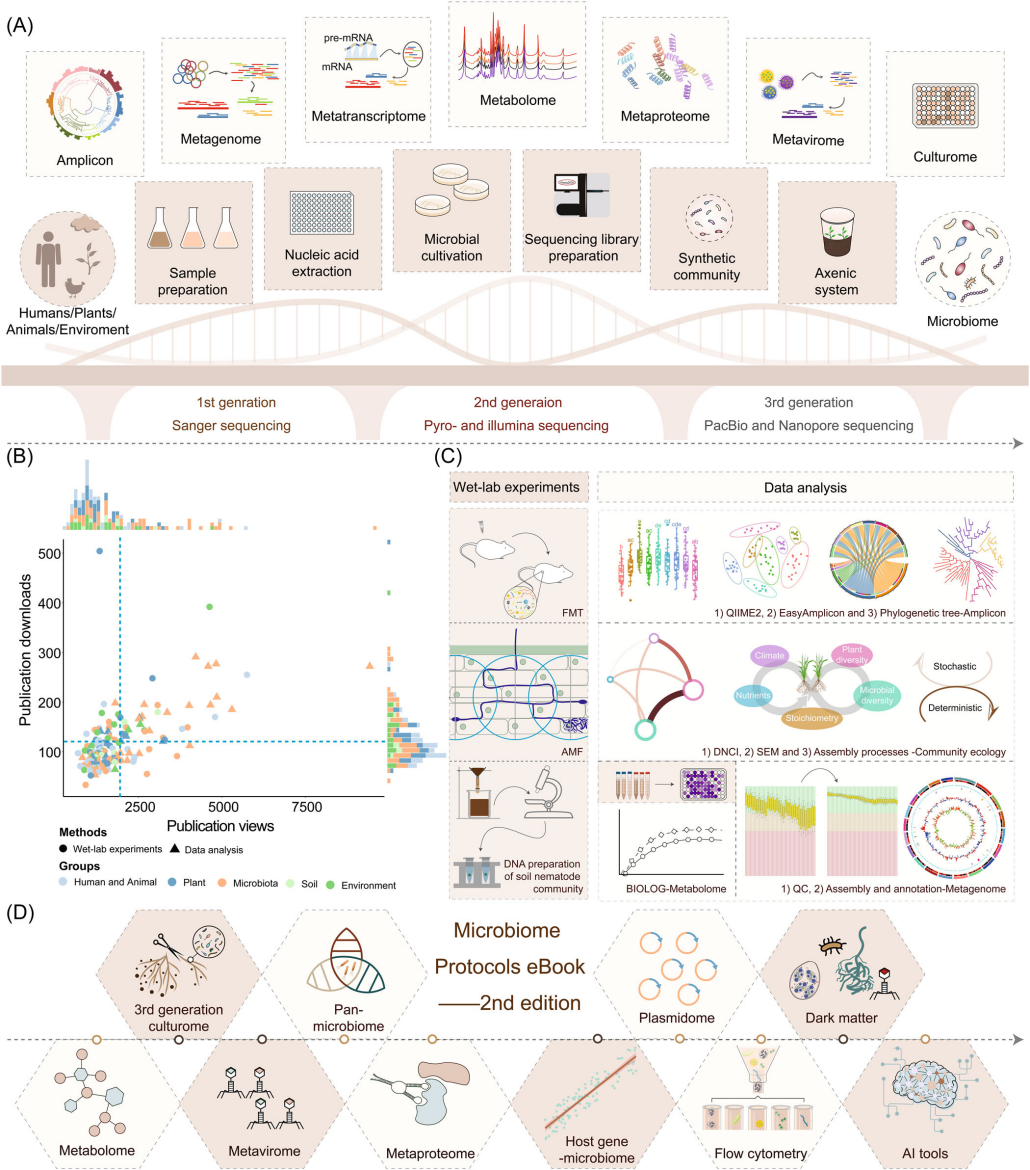
Microbiome Protocols eBook (MPB)—Building a bridge to microbiome research. The establishment and optimization of a variety of microbiome protocols make it possible to study the microbiome of humans, animals, plants, and the environment. (A) Sequencing technology, wet‐lab experiments, and data analysis methods link the microbiome with its host or environment. (B) The publication views and downloads of each protocol published in MPB, until 19th January 2024. (C) The 12 most popular publications in MPB. (D) The 10 key recruitment focuses for the 2nd edition of the MPB. Vector for the 3rd generation culturome is modified from Zhang et al. [9]. AMF, arbuscular mycorrhizal fungi; DNCI, dispersal‐niche continuum index; FMT, fecal microbiota transplantation; QC, quality control; SEM, structure equation modeling.

Since the first announcement of MPB in July 2020, 355 researchers from 125 institutes or universities have been involved in the MPB, including the Institute of Genetics and Developmental Biology, Institute of Soil Science, Institute of Microbiology, Research Center for Eco‐Environmental Sciences, and Institute of Urban Environment of Chinese Academy of Sciences, Chinese Academy of Agricultural Sciences, Peking University, Tsinghua University, Zhejiang University, Sun Yat‐Sen University, China Agricultural University, Shandong University, Yangzhou University, Westlake University, Nanjing Agricultural University, and so forth. As of January 19th, 2024, 1st edition of MPB has published 152 protocols, with an average of 1945.47 pageviews and 210.39 downloads in per protocol. Over the past year, microbiome data analysis has been particularly popular among readers (Figure 1B). Upon summarizing both pageviews and downloads data, we identified 12 most popular works, consisting of 3 wet‐lab experiments and 9 data analysis works (Figure 1C, Supporting Information: Table S2). Notably, in previous endeavors, the mainstream focus remained on the data analysis of amplicon and metagenome, primarily due to the maturity of second‐generation sequencing technology.

Of the 12 notable contributions highlighted in the 1st edition of MPB, several have significantly propelled research into microbiome analysis. For instance, the wet‐lab experiment protocol of soil nematode community [11] has addressed the limitations of available nematode sequences, offering a standardized approach for studying soil nematode community using high‐throughput techniques. Since then, it has garnered more than 3000 views and has facilitated the publication of five studies exploring the diversity or composition of soil communities. EasyAmplicon [10] has emerged as a widely utilized tool for amplicon data analysis. Since its publication in our protocol, it has accumulated over 4000 views. Our protocol serves as an interactive platform for both authors and users, fostering the continual improvement of the tool. To date, EasyAmplicon has supported 100 publications for mining data, spanning the microbiome in animal, soil, waste water, plant, wine, and so forth.

To ensure the quality, diversity, and timeliness of the MPB, we have established it as a long‐term project with biennial updates. In the upcoming 2nd edition of MPB, we are excited to incorporate updates from the 1st edition's protocols while also expanding into new areas, covering innovative methodologies and emerging technologies. This includes aspects such as the 3rd generation culturome, pan‐microbiome [12], metabolome [13], metavirome, metaproteome, plasmidome, the “dark matter” of the microbiome [14], and the interaction of host genetics with the microbiome (Figure 1D). Additionally, we will feature cutting‐edge technologies and data algorithms, including flow cytometry and AI tools, such as machine learning and deep learning algorithms (Figure 1D). The published protocols can be accessed on the project homepage. We sincerely invite more researchers to participate in this project and contribute to their protocols. Any protocols related to the microbiome are welcome, especially for the commonly used or cutting‐edge protocols related to the 10 key focuses (Figure 1D). We hope MPB becomes a protocol encyclopedia and a valuable tool for microbiome research.

## AUTHOR CONTRIBUTIONS

Yong‐Xin Liu, Yang Bai, and Haiyan Chu conceived and coordinated this work. Yunyun Gao and Kai Peng authored the paper, and the other authors have revised the manuscript. All authors have read the final manuscript and approved it for publication.

## CONFLICT OF INTEREST STATEMENT

The authors declare no conflict of interest.

## Supporting information


**Table S1**. The publication views and downloads of each protocol published in the first edition of MPB, until 19th Jan, 2024.
**Table S2**. The 12 most popular publications in the first edition of MPB.

## Data Availability

The data that supports the findings of this study are available in the supplementary material of this article. All the protocols are open access in https://bio-protocol.org/bio101/mpb. Supplementary materials (tables, graphical abstracts, slides, videos, Chinese translated version and update materials) may be found in the online DOI or iMeta Science http://www.imeta.science/.
